# Assembly process and co-occurrence network of microbial community in response to free ammonia gradient distribution

**DOI:** 10.1128/spectrum.01051-24

**Published:** 2024-07-26

**Authors:** Shengjie Sun, Zhiyi Qiao, Kexin Sun, Da Huo

**Affiliations:** 1 Tianjin Key Laboratory of Aqua-Ecology and Aquaculture, College of Fisheries, Tianjin Agricultural University, Tianjin, China; 2 Frasergen Bioinformatics Co., Ltd, Wuhan, China; 3 CAS Key Laboratory of Algal Biology, Institute of Hydrobiology, Chinese Academy of Sciences, Wuhan, China; Fujian Agriculture and Forestry University, Fuzhou City, Fujian, China

**Keywords:** network analysis, community assembly, free ammonia, microbial community

## Abstract

Microorganisms are crucial components of the aquatic ecosystem due to their immense diversity and abundance. They are vital in sustaining ecological services, especially in maintaining essential biogeochemical cycles. Recent years have seen a substantial increase in surplus nitrogenous pollutants in aquatic ecosystems due to the heightened occurrence of anthropogenic activities. Elevated levels of free ammonia (FA, NH_3_), stemming from the discharge of excess nitrogenous pollutants, have caused notable fluctuations in aquatic ecosystems, leading to water eutrophication and various ecological challenges. The impact of these oscillations on microbial communities in aquatic ecosystems has not been extensively studied. This study employed 16S rRNA gene amplicon sequencing to systematically investigate the dynamics, co-occurrence networks, and assembly processes of microbial communities and their subcommunities (abundant, moderate, and rare) in the Luanhe River Diversion Project in China. Our findings indicate that NH_3_ concentration significantly influences the dynamics of microbial communities, with a notable decrease in community Richness and Phylogenetic Distance alongside increased community dissimilarity under higher NH_3_ conditions. The analysis revealed that certain microbial groups, particularly Actinobacteriaota, were notably more prevalent in environments with elevated NH_3_ levels, suggesting their potential resilience or adaptive responses to NH_3_ stress. Additionally, through co-occurrence network analysis, we observed dynamic changes in network topology and increased connectedness under NH_3_ stress. Key nodes, identified as connectors and module hubs, played crucial roles in maintaining network structure, particularly Cyanobacteria and Actinobacteriaota. Furthermore, stochastic processes, particularly drift and dispersal limitation, predominantly shaped the microbial communities. Within the three subcommunities, the impact of drift became more pronounced as the effect of dispersal limitation diminished. Overall, elucidating the dynamics of microbial communities in aquatic ecosystems exposed to NH_3_ can enhance our comprehension of the ecological mechanisms of microbial communities and provide new insights into the conservation of microbial community diversity and ecological functions.

**IMPORTANCE:**

The research presented in this paper explores how varying concentrations of free ammonia impact microbial communities in aquatic ecosystems. By employing advanced gene sequencing techniques, the study reveals significant changes in microbial diversity and network structures in response to increased ammonia levels. Key findings indicate that high ammonia concentrations lead to a decrease in microbial richness and diversity while increasing community dissimilarity. Notably, certain microbial groups, like Actinobacteria, show resilience to ammonia stress. This research enhances our understanding of how pollution affects microbial ecosystems and underscores the importance of maintaining balanced ammonia levels to preserve microbial diversity and ecosystem health.

## INTRODUCTION

The heightened occurrence of anthropogenic activities, such as urbanization, industrialization, large-scale agricultural expansion, and aquaculture, has resulted in the significant release of nutrients and pollutants, including ammonia nitrogen, into aquatic systems. These inputs are further exacerbated by environmental factors like stormwater runoff, atmospheric emissions, and sedimentation (1, 2). At the same time, the Free Ammonia (FA, NH_3_), as a form of ammonia nitrogen present in water, has risen in response to the discharge of surplus nitrogenous pollutants (3). Free Ammonia (FA, NH_3_) and ionized-ammonia (NH_4_^+^-N) are the two forms of ammonia nitrogen present in natural water, depending on the pH and temperature of the water. The equilibrium between these forms is disrupted, leading to an increase in the concentration of NH_3_ when both temperature and pH rise. Increases in global temperatures are likely to disrupt existing equilibria, potentially resulting in elevated concentrations of the more toxic form of Free Ammonia. This temperature-driven escalation in NH_3_ concentration is posited to significantly amplify its toxicity, based on the understanding of chemical equilibria under varying thermal conditions (4). Consequently, this has triggered pronounced fluctuations in the environmental state of aquatic ecosystems, resulting in water eutrophication and a chain of ecological issues, resulting in changes in microbial community dynamics (5).

Microorganisms have a crucial role in maintaining the major biogeochemical cycles, which are essential for supporting other life forms and ecological services (6). Moreover, nitrogen biogeochemical processes, predominantly regulated by microorganisms, are vital for the nutrient cycling of water (7). FA has the capacity to impose inhibitory and/or biocidal effects, and its concentration is primarily affected by the pH and temperature of the water. Increased FA concentrations not only inhibit the function of ammonia-oxidizing bacteria responsible for nitrification of nitrogen cycling but also lead to a reduction in entire microbial community diversity (8, 9). This inhibition may reduce microbial cell activity and enzymatic functions, potentially leading to a reconfiguration of the microbial community (10). Therefore, understanding the dynamics of microbial communities in aquatic ecosystems, especially under FA stressors, can improve our knowledge of their ecological mechanisms and aid in preserving their diversity and functions.

Additionally, previous studies have shown that microbial community composition and abundance in aquatic ecosystems are unevenly distributed (11, 12). A few species constituted the majority of the abundance, whereas most species contributed minimally to the overall abundance (13). Dominant microbes comprised the majority of microbial biomass and played key roles in ecological functions, forming the “abundant subcommunity” (14). Conversely, low-abundance microbes, which constitute the majority of local species, offer diverse ecological services and ensure functional redundancy and flexibility, defining the “rare subcommunity” (15). As mentioned, the “moderate subcommunity” also contributes to community stability and performs various ecological services. It is noteworthy that subcommunities show varied responses to environmental stress, mainly due to differences in their abundance and diversity. A study demonstrated that microbial subcommunities adopt distinct strategies to cope with environmental stress. Abundant subcommunities dominate the taxonomic composition, whereas rare subcommunities influence the functional composition. Under environmental stress, the taxonomic diversity of abundant subcommunities increased but that of rare subcommunities remained limited (16).

Furthermore, applying network analysis to taxa co-occurrence patterns offers fresh insights into the structure of microbial communities and has garnered growing attention in microbial ecology research (17). The interactions of microorganisms like competition, predation, mutual symbioses, and trade-offs can be interpreted through the topological properties of the co-occurrence network, particularly the relationships between nodes and edges (18). The complex interactions among microorganisms drive community dynamics and impact ecosystem function and stability. A decline in the complexity and stability of co-occurrence networks of the microbial community may pose a threat to community diversity and ecological function (19, 20). Meanwhile, the assembly process of the microbial community is a pivotal topic in ecological research (12, 21). Stochastic and deterministic processes serve as two fundamental and complementary mechanisms in the assembly process (22, 23). Deterministic processes are tied to selection, emphasizing factors like species characteristics, interspecific interactions, and environmental conditions that control community structure. This is often termed the “niche-based theory.” Stochastic processes involve drift (DR), dispersal, and diversification, emphasizing that community structures are independent of species traits and are shaped by random processes such as birth, death, colonization, extinction, and speciation. This is commonly known as the “neutral theory” (24). A study (25) revealed differences in bacterial interactions within the co-occurrence network of bacteria due to changes in hydrological and environmental conditions due to seasonal variations. Bacterial interactions are weaker during the wet season, whereas they are stronger during the dry season. The assembly process of abundant communities is mainly influenced by the dispersal limitation (DL) of stochastic processes, and the rare communities are mainly influenced by heterogeneous selection (HeS) of deterministic processes. Therefore, revealing the distinct strategies of subcommunities under environmental stress is necessary to help us better maintain the stability of aquatic ecosystems. Research on the effects of fluctuating free ammonia levels on the co-occurrence networks and assembly processes of microbial communities in aquatic ecosystems remains sparse yet essential.

In this study, we employed high throughput 16S rRNA sequencing in conjunction with environmental variables to investigate variations in the composition and diversity of microbial communities across the Luanhe River Diversion Project in China, including the Yuqiao, Panjiakou, Daheiting reservoirs, along with their tributaries, and the Luan River basin in Tianjin, northern China. Our study aims to address the following questions: (i) how does the structure and diversity change in distinct microbial subcommunities under the stress of environmental conditions such as the FA? (ii) What alterations occur in the dynamics of co-occurrence networks and stability under the influence of FA? (iii) Are assemblage processes of abundant, moderate, and rare subcommunities dominated by stochastic or deterministic factors?

## MATERIALS AND METHODS

### Sample collection and environmental attributes analysis

In August, September, and October 2021, 35 water samples were collected from 12 sites (Fig. S1) within the Luanhe River Diversion Project in Tianjin, northern China. This included the Yuqiao, Panjiakou, and Daheiting reservoirs, along with their tributaries, and the Luan River basin. This project connects the Panjiakou, Daheiting, and Yuqiao reservoirs through north and south diversion lines, forming a vital water supply system for northern China and providing water resources to cities such as Tianjin and Beijing. The sampling sites, located in a temperate monsoon climate zone, have their specific coordinates listed in Table S1. Surface water (500 mL) from each sampling site was immediately filtered using a 0.22 µm polycarbonate membrane (Millipore, USA). The environmental variables, including water temperature, salinity, Siemens, pH, dissolved oxygen, nitrate-nitrogen (NO_3_^-^N), Free ammonia (NH_3_), ammonium-nitrogen (NH_4_^+^-N), and chlorophyll, were promptly measured at the sampling sites using the YSI 650 MDS Multimeters (YSI, USA). It needs to be added that ammonium (NH_4_^+^-N) is measured using a silver/silver chloride wire electrode through an ion-selective membrane, which establishes a potential based on the ammonium concentration, described by the Nernst equation. The system calculates free ammonia (NH_3_) concentrations using concurrent pH, temperature, and conductivity measurements to determine the equilibrium between NH_4_^+^-N and NH_3_. The environmental attributes of each sample are listed in Table S1.

### DNA extraction, PCR amplification, and sequencing

The microbial DNA was extracted using the Magen HiPure Soil DNA Kit (Magen, Guangzhou, China). The concentration of the extracted DNA was measured using the Qubit fluorometer (Thermo Scientific, USA), while the purity was assessed using the Nanodrop 2000 (Thermo Scientific, USA).

For amplification of the hypervariable region V3–V4 of the bacterial 16S rRNA gene, the primer pair 338F/806R was employed. The PCR products were initially evaluated through agarose electrophoresis (1%) and subsequently purified using the QIAquick PCR Purification Kit (Qiagen, Germany). The purified PCR products were then pooled in equal amounts and sequenced on the Illumina NovaSeq PE250 platform at Frasergen (Wuhan, China).

### Sequencing data processing

The raw FASTQ files underwent a series of standard processing procedures using QIIME2 v2023.2 (26). The paired-end reads were merged and quality-filtered using VSEARCH with default parameters (27). The DADA2 pipeline was then employed for denoising and generating amplicon sequence variants (ASVs) (28). To determine the taxonomic information for each ASV, the Silva 16S rRNA database v138.1 was utilized (29). To ensure comparability, the microbial profiles were rarefied to the same sequencing depth using the “Rarefy” function of the “GuniFrac” package in R v4.2.1 (30). Subsequently, all further analyses were conducted in R, unless otherwise specified.

In this study, the abundant and rare bacterioplankton communities were clarified following a previous method (17, 25, 31). The relative abundances exceeding 0.1% were classified as abundant taxa, while those below 0.01% were considered rare taxa. Those within the range between these thresholds were categorized as moderate taxa.

### Statistical and ecological analysis

The α-diversity index (Shannon, Simpson, and Richness) for each sample and the β-diversity index (“Bray-Curtis” distance) for each pairwise sample both using the “vegan” package. The sum of the total phylogenetic branch length (phylogenetic distance, PD) was calculated using the “picante” package (32). To examine the relationships between physicochemical properties and the microbial community structure, Mantel tests were performed. Spearman correlation was used to determine the correlation between environmental factors and typical taxa. Linear regressions were employed to investigate the changes in α-diversity and taxonomic composition. The analysis of changes in β-diversity with NH_3_ was conducted through a linear regression between the “Bray-Curtis” similarity distance and Euclidean distance of NH_3_. The geographic distance between each pairwise sampling site was calculated using the “geosphere” package, considering the longitude and latitude data. The distance-decay relationship was determined by calculating the slope of an ordinary least squares regression between geographic distance and “Bray-Curtis” similarity.

Data visualization is performed by the “ggplot2” and the “ggpubr” packages, and partial figure color matching is provided by the “RColorBrewer” package. *R*^2^ and *P* values are displayed in the figure, using the “ggpmisc” package.

### Ecological network analysis

To investigate the dynamic of microbial co-occurrence networks with the NH_3_, we divided the sample into four groups according to the order of their NH_3_ levels (n1–n4, with average NH_3_ ranging from 0.11 to 1.07). The integrated Network Analysis Pipeline (INAP) platform (http://mem.rcees.ac.cn:8081/) was utilized to construct the microbial co-occurrence networks (33). The SparCC method (34) was used to construct the microbial co-occurrence networks, considering the sparsity of microbiome data sets. Meanwhile, the ASV appearing only once in the sample was filtered out, and each group contained at least eight samples. To ensure the robustness of the networks, the following parameters were set: the number of inference iterations to average over was set to 20, while the number of exclusion iterations to remove strongly correlated pairs was set to 10. A correlation strength exclusion threshold of 0.1 was applied. Furthermore, 100 shuffling iterations were performed. The computation of *P*-values was conducted using a two-sided test, resulting in the generation of SparCC Correlation Coefficient matrixes.

To ameliorate the problems of ill-conditioning, self-looping, and interaction strength overflow of networks, as well as to improve the accuracy of network prediction, the Inference of Direct and Indirect Relationships with Effective Copula-based Transitivity method (35) is used to filter the nodes and edges of the network for quantitatively inferring direct dependencies in association networks. For the construction of the networks, a uniform threshold was applied to ensure comparability. The correlation coefficient threshold used for constructing the networks was an *R*-value higher than 0.6 and a *P*-value less than 0.01. The topological analysis of the network was using the INAP platform, and the visualization was carried out using the Gephi v0.10.1 (36)

Module separation and modularity calculation were performed using greedy modularity optimization, and the within-module connectivity (Zi) and among-module connectivity (Pi) values for all nodes were calculated (37). With certain assignment criteria, the nodes could be classified into network hubs, module hubs, connectors, and peripherals. Network hubs were *Z* larger than 2.5 and *P* larger than 0.62 (highly connected nodes within the entire network), module hubs were *Z* larger than 2.5 and *P* less than 0.62 (highly connected nodes within modules), connectors were *Z* less than 2.5 and *P* larger than 0.62 (nodes that connect modules), peripherals were *Z* less than 2.5 and *P* less than 0.62 (nodes connected in modules with few outside connections). To assess the stability of the networks under species extinction scenarios, simulations were conducted by randomly removing a certain proportion of nodes in each network (38). The average degree and natural connectivity were then computed to evaluate network stability.

### Inferring bacterial community assembly mechanisms

The neutral community model assessed the influence of stochastic processes on the microbial community assembly by predicting the detection frequency and relative abundance relationship for each ASV (39). The “MicEco” package was used to calculate the fit (*R*^2^) of the neutral community model, with a positive *R*^2^ indicating a good model fit. The ICAMP (Infer Community Assembly Mechanisms by Phylogenetic bin-based null model analysis) model was employed to quantify the mechanisms of community assembly, determining the influence of various ecological processes on community structure (40). The phylogenetic tree is used to divide multiple phylogenetic bins. Each bin that is governed by the ecological process is identified through null model analysis of two metrics: the phylogenetic metric βNRI (beta Net Relatedness Index) and the taxonomic β-diversity metric RC (modified Raup-Crick metric). Five different processes are identified for each bin: homogeneous selection (HoS), HeS, DL, homogenizing dispersal (HD), and DR. For each bin, the percentages of HoS are determined when βNRI < −1.96, and the percentages of heterogeneous selection are calculated when pairwise βNRI > 1.96, which indicated significantly high or less phylogenetic turnover than expected, respectively. Subsequently, the RC metric based on taxonomic diversity is used to further segment the remaining pairwise comparisons with |βNRI| ≤ 1.96. The fraction of pairwise comparisons with RC < −0.95 is treated as the percentages of HD, while those with RC > 0.95 as dispersal limitation, implied significant deviations from null model expectations. The remaining comparisons with |βNRI| ≤ 1.96 and |RC| ≤ 0.95 represent the percentage of drift or undominant process. The analysis of the above was performed using the R package “iCAMP” with the function “icamp.big.” A total of 999 random permutations were conducted, while “ses.cut” was set to 1.96, “rc.cut” to 0.95, and “bin.size.limit” to 12. Default values were used for other parameters.

## RESULTS

### Dynamics of bacteria community composition and diversity on the free ammonia stress

We collected a total of 35 samples from the Luanhe River Diversion Project and performed total DNA extraction. In each sample, we amplified and sequenced the V3–V4 region of the 16S rRNA gene. Subsequently, we processed the data, which included quality filtering, chimera removal, and rarefaction. We retained 23,151 merged sequences per sample, which were clustered into 13,241 ASVs. Taxa with relative abundances exceeding 0.1% were classified as abundant, while those below 0.01% were considered rare. Taxa falling between these thresholds were categorized as moderate. Specifically, we identified 95 ASVs, accounting for 15.12% of the relative abundance, as abundant taxa, 2,411 ASVs, making up 58.86% of the relative abundance, as moderate taxa, and 10,735 ASVs, representing 26.02% of the relative abundance, as rare taxa.

**Fig 1:**
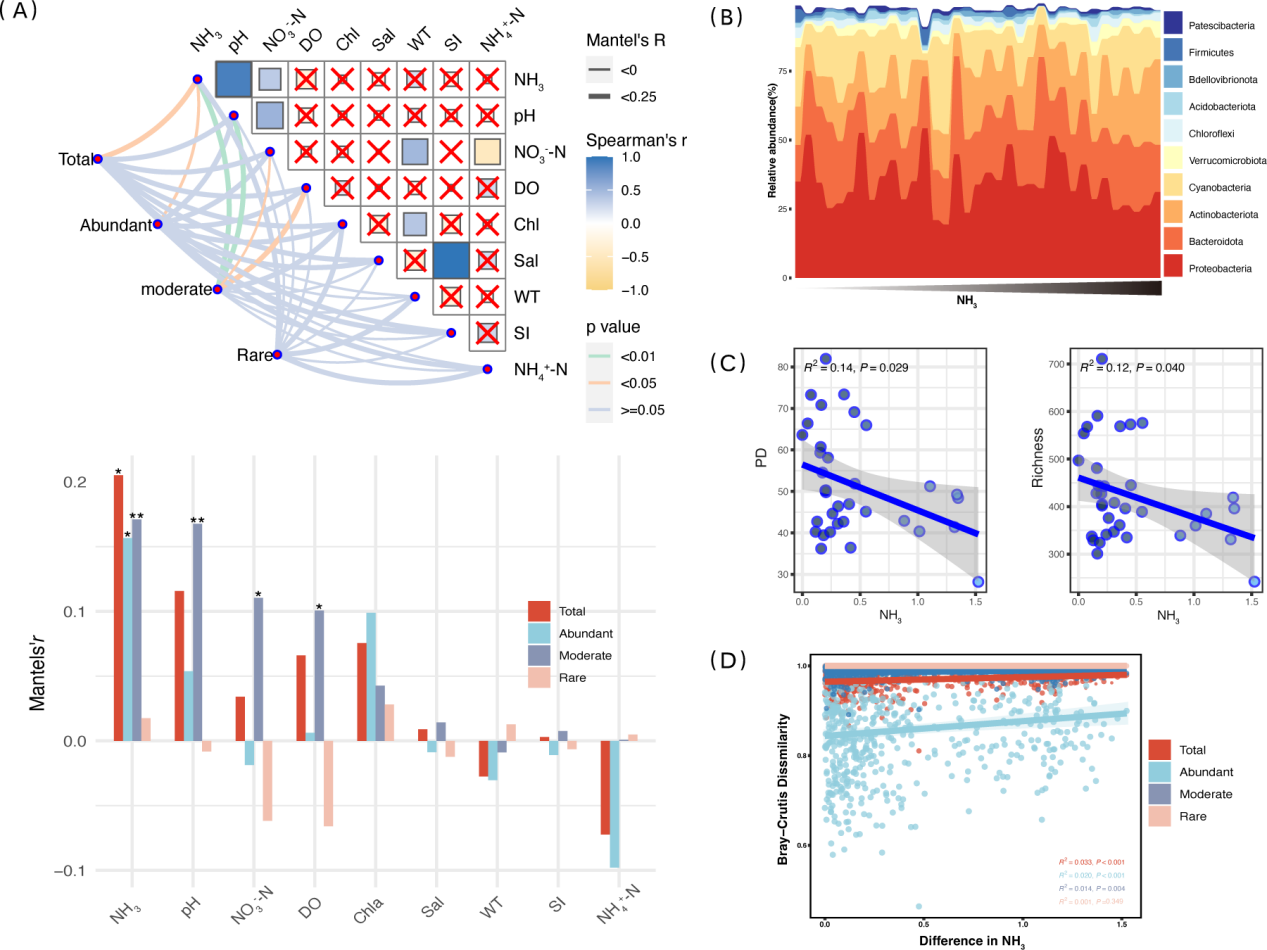
Shifts in the diversity and composition of the microbial community under the NH_3_ stress. (**A**) Mantel test of environmental drivers of the microbial community and its subcommunities (*P < 0.05 and **P < 0.01). (**B**) Taxonomic composition of the microbial community at the phylum level under the NH_3_ gradient, only the top ten phyla. (**C**) Trends in the diversity of the microbial community with NH_3_. (**D**) Linear regression between the “Bray-Curtis” similarity distances of community and the Euclidean distances of NH_3_.

The environmental drivers of the microbial community were analyzed using the Mantel test. The result revealed that the NH_3_ (FA) had a significant impact on the total microbial community, as well as on the abundant subcommunity (*P* < 0.05). The moderate subcommunity was highly significantly affected by pH and NH_3_ (*P* < 0.01). Additionally, DO and NO_3_^-^N also had an impact on the moderate subcommunity (*P* < 0.05, Fig. 1A). Among these environmental factors, NH_3_ exhibited the highest Mantel’s *r* value, signifying that it was the most influential factor associated with the structures of the entire community, including the abundant and moderate subcommunities (Fig. 1A). Further analysis of the effect of NH_3_ on the composition of the bacterial community showed that the taxonomic classification of these representative ASVs included that the bacteria taxa from 45 phyla, 131 classes, 331 orders, 554 families, and 1,094 genera. Among the ten most abundant microbial phylum, the bacterial community was dominated by Proteobacteria (34.74%), Bacteroidota (22.15%), Actinobacteria (17.58%), Cyanobacteria (14.74%), and Verrucomicrobiota (2.65%); these top five phyla constituted 77.19% of the total ASVs. It is also noteworthy that the Actinobacteriaota exhibited a very significant increase with the rise of NH_3_ (*P* < 0.01, Fig. 1B). Additionally, species abundance with significant changes in free ammonia levels was analyzed using Spearman correlation. The results indicated that 36 species were either significantly (*P* < 0.05) or very significantly (*P* < 0.01) correlated with free ammonia. These species represented 2.89% of the total abundance. Among them, 11 were abundant species and 25 were moderate species; nine exhibited a negative correlation with NH_3_, while 27 were positively correlated. The species were classified as 16 Proteobacteria, 7 Actinobacteriota, 6 Cyanobacteria, and 3 each from Bacteroidota and Verrucomicrobiota (Fig. S2).

Furthermore, the linear regression analysis revealed a significant decrease in PD and Richness with increasing NH_3_ levels (Fig. 1C, *P* < 0.05). However, the Simpson and Shannon indices remained unaffected (Fig. S3, *P* > 0.05). Based on correlation analysis between each pairwise samples “Bray-Curtis” similarity distances and the Euclidean distances of NH_3_, both the total and abundant communities exhibited a highly significant distribution pattern (*P* < 0.001). Community dissimilarity increased with rising NH_3_ levels. Similar patterns were also observed in the moderate community (Fig. 1D, *P* < 0.01). Nonetheless, both the microbial communities and their sub-communities exhibited no significant spatial variation in distance decay patterns (Fig. S4, *P* > 0.05).

### Co-occurrence pattern of bacterial community

**Fig 2:**
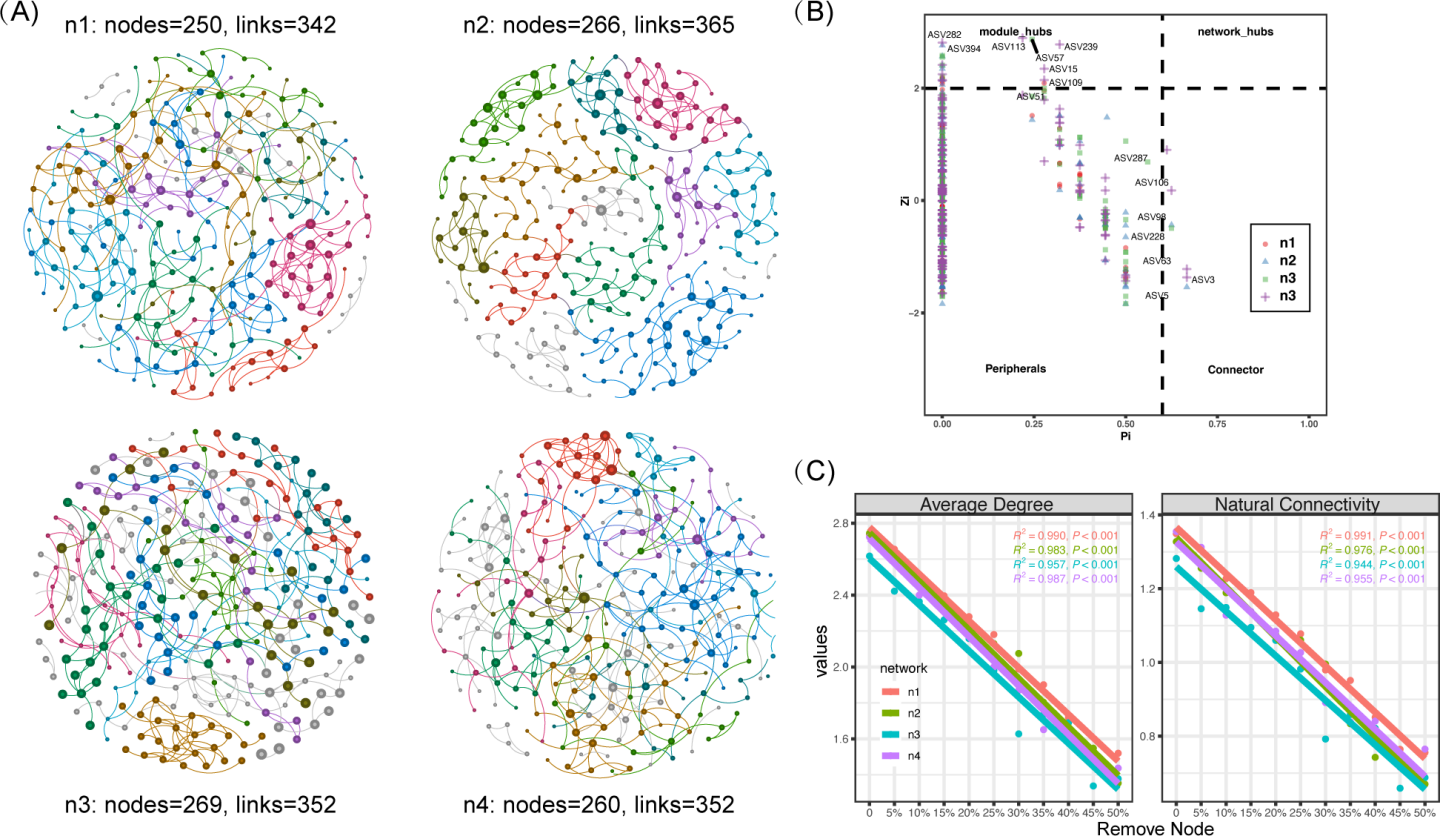
Network analysis of microbial community under the NH_3_ gradient. (**A**) The co-occurrence networks of the microbial community. Networks were constructed based on their NH_3_ levels, labeled as n1–n4, with an average NH_3_ range of 0.11 to 1.07. Nodes and links in the networks are colored based on module attributes. The top 10 largest modules have distinct colors, while the remaining smaller modules are shaded gray. Node size corresponds to its degree. (**B**) The key nodes in the networks are selected by within-module connectivity (Zi) and among-module connectivity (Pi). (**C**) Network stability was evaluated based on average degree and natural connectivity following species extinction, achieved by proportionally removing nodes at random.

We constructed four microbial ecological networks by processing SparCC correlation coefficients with the IDRECT method, aiming to elucidate the dynamic changes in microbial associations with NH_3_ (Fig. 2A). These four networks comprised 250–269 nodes connected by 342–365 links, with the majority of these links (95.19%–98.57%) being positive. Among these networks, n2 exhibited the highest average degree (2.74). Regarding the average clustering coefficient, which measures the degree to which nodes in the network cluster together, n1 had the highest value (0.086), while n3 had the lowest (0.068). It is important to emphasize that connectedness, an indicator used to evaluate the extent of connectivity among all nodes in the network, exhibits a rising trend in response to free ammonia pressure, increasing from 0.32 to 0.83 (Table S2).

Additional investigation was carried out to examine the topological roles of nodes in the four networks. Nodes were categorized based on their within-module connectivity (Zi) and among-module connectivity (Pi) values. Module hubs and connectors are frequently denoted as key nodes (species) that have a crucial impact on shaping the structure of ecological networks and upholding network stability. A total of 7 connectors and 31 module hubs were identified in the four networks (Fig. 2B). The seven connectors belonged to various taxonomic groups, including two from Actinobacteriota (g_*hgcI clade* and g_*CL500-29 marine group*), two from Protecobacteria (g_*Limnohabitans*. and *g_Sphaerotilus*), two from Bacteroidota (*f*_*NS9 marine group* and *f_NS11-12 marine group*), and others to Verrucomicrobiota. It is worth noting that no connectors were identified in the n1 networks. Module hubs were also detected in the four networks, with 11 module hubs primarily consisting of Cyanobacteria, 10 module hubs from Actinobacteriota, and the remaining hubs from Bacteroidota, Chloroflexi, Verrucomicrobiota, and Proteobacteria (Table S3).

Furthermore, species extinction was simulated by randomly removing nodes in proportion, and the network’s stability was assessed using the average degree and the natural connectivity index. The findings revealed that as network nodes were removed in proportion, the network’s stability decreased. However, it is noteworthy that the n1 network exhibited higher robustness compared to all other networks (Fig. 2C Table S4).

### Assembly process of bacterial community

**Fig 3:**
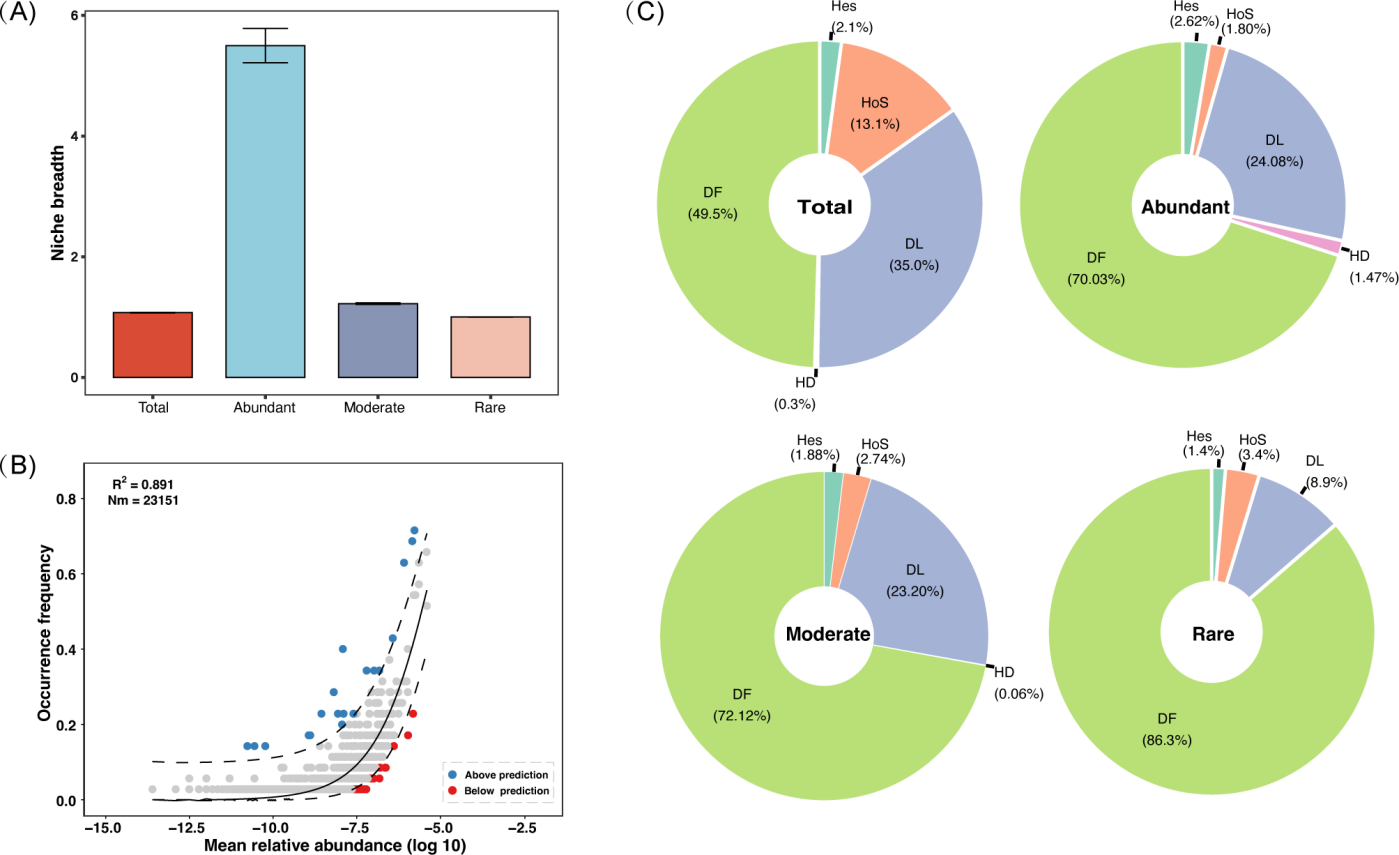
The niche breadth of the microbial community and the contributions of various ecological processes to the microbial community. (**A**) The niche breadth of the microbial community. (**B**) Fit of the neutral community model. The blue and red circles represented ASVs that occurred more and less frequently than predicted, respectively. The solid black line donated the best fit to the neutral community model, and the dashed black lines represented the 95% confidence intervals. Nm was the product of metacommunity size and migration rate, and *R*^2^ represented the model’s fit. (**C**) Details the contributions of five distinct ecological processes to the microbial community: heterogeneous selection (HeS), homogeneous selection (HoS), dispersal limitation (DL), homogenizing dispersal (HD), and drift (DF).

The niche breadth was calculated using Levins’ standardized niche breadth index, the abundant community has a much higher niche breadth of 5.49. The total and moderate communities were lower, at 1.07 and 1.22, respectively. The lowest is the rare sub-community with almost 1 (1.0018; Fig. 3A). Furthermore, when applied to the total microbial community assembly, the neutral community model displayed a high fit (*R*^2^ = 0.891), with only 0.52% of taxa deviating from its predictions, indicating a predominance of stochastic processes in the total community assembly (Fig. 3B).

The null model quantified community assembly mechanisms, assessing the impact of different ecological processes on community structure. Based on βNRI and RCbray values, five different ecological processes (heterogeneous selection, homogeneous selection, dispersal limitation, homogenizing dispersal, and drift) were classified to quantify the compositional variations of microbial communities. Stochastic processes dominate the assembly of the total community, with drift being the primary driver of changes in bacterial community composition (49.5%). It is followed by dispersal limitation (35.0%), homogeneous selection (13.1%), heterogeneous selection (2.1%), and homogenizing dispersal (0.3%; Fig. 3C). Moreover, slightly similar assembly processes of subcommunity were observed for abundant, moderate, and rare, respectively. The same stochastic processes dominate the assembly of sub-communities, and stochastic processes dominate the assembly of subcommunities, with drift being the primary driver of structural changes in the subcommunities, accounting for 70.03%, 72.12%, and 86.3%, respectively. Dispersal limitation also influences the subcommunities, with contributions of 24.08%, 23.20%, and 8.9%, respectively. It is important to highlight that from rich to rare communities, drift increases by 16.27%, while dispersal limitation decreases by 15.18%.

## DISCUSSION

### Response of free ammonia on microbial community dynamics

Excessive ammonia nitrogen inputs from anthropogenic sources pose a significant threat to water quality and can lead to ecological disasters, resulting in changes in microbial community dynamics (41, 42). In our study, using the common thresholds for classifying abundant and rare species (0.1%–0.01%), we found that 95 ASVs were categorized as abundant, accounting for 15.12% of the relative abundance; 2,411 ASVs were moderate, making up 58.86% of the relative abundance; and 10,735 ASVs were rare, representing 26.02% of the relative abundance. While these results appear to contradict the traditional understanding that abundant species dominate the majority of the community’s relative abundance, we do not believe this challenges traditional theory. We utilized ASV clustering with 100% sequence similarity rather than the traditional operational taxonomic units with a 97% similarity threshold. This higher resolution allows for more precise differentiation of species, resulting in a greater number of distinguishable taxa and a reduction in the relative abundance of single species, leading to a higher count of moderately abundant species. ASV clustering provides a more accurate representation of microbial community composition, revealing greater diversity within samples, including those with moderate abundance (43). Meanwhile, our findings indicate that NH_3_ levels influence microbial community dynamics. Elevated NH_3_ concentrations led to reductions in both richness and PD indices across the community, while the Simpson and Shannon indices remained relatively stable. This means that community species richness and evolutionary diversity were suppressed under NH_3_ stress. Although NH_3_ elevation may cause a decline in certain species due to selective pressures, it does not necessarily signify a notable change in the relative abundance among the remaining species. In other words, despite the reduction in species count, the species distribution within the ecosystem remains relatively even, exhibiting a decoupling state of species richness and species evenness (44). This may be related to the redundancy of community functions. Alternatively, microbial communities might exhibit resilience and adaptability, responding to NH_3_ elevations without major shifts in species evenness. Certain species might have adapted to elevated NH_3_ levels, ensuring the preservation of ecosystem balance (10, 45). Adaptation to stress, both at the community and individual levels, contributes to the stability and resilience of aquatic ecosystems (46). In prior research on marine and wastewater environments, a negative correlation was observed between free ammonia and microbial community diversity (47, 48). Meanwhile, the microbial community largely regulates nitrogen biogeochemical processes, and free ammonia stress can impact ecological services functions such as nitrogen cycling pathways and rates in rivers (7, 49, 50).

Furthermore, our research has found that the Spearman correlation analysis highlighted that those nine species had a significant negative correlation with free ammonia, while 27 species showed a positive one. Such results suggest that free ammonia may suppress certain sensitive species. Conversely, species adept at utilizing free ammonia or those with a higher intracellular pH seem to have a competitive advantage (51, 52). The biotoxicity of free ammonia seems to diminish Richness in communities at sites with higher ammonia concentrations. This might escalate community variability or turnover, subsequently modifying community structure and increasing Bray-Curtis dissimilarity distances. In essence, free ammonia’s impact on specific bacterial abundance, diversity, and community composition might lead to a disrupted microbial community structure, potentially compromising ecological functionality and ecosystem stability (53, 54)

### Microbial co-occurrence networks under free ammonia stress

In the co-occurrence network analysis, we observed dynamic changes in network topology and increased connectedness under NH_3_ stress (0.33–0.83). This is consistent with previous research that environmental stresses may cause a rise in network connectivity (55). The stressed microbial communities exhibit lower network stability, with increased connectivity between modules potentially acting as a compensatory mechanism. The increased connectivity may reflect adaptive changes in the ecological network in the face of environmental stresses from elevated NH_3_ concentrations. This increased internal connectivity may help the network to functionally resist perturbations and maintain the continuity of ecological processes. While increased connectivity may help stabilize microbial communities in the short term, overdependence on specific microbial interactions may make the network more sensitive to future environmental changes (56). This dependence may affect community resistance and resilience in the long term. In addition, reduced species diversity may lead to a simplification of ecological functions despite increased network connectivity (17). Reductions in species diversity are often associated with declines in ecological services, and thus, the functional stability of the overall ecosystem may be threatened, even if the network structure shows increased resilience.

We conducted further analysis to investigate the topological roles of key nodes in the four networks, based on their within-module connectivity (Zi) and among-module connectivity (Pi) values. Cyanobacteria and Actinobacteriota emerged as the major key hubs in all four networks. As noted earlier, the prevalence of Actinobacteriaota significantly increased with rising NH_3_ levels (*P* < 0.01). Some strains within this group demonstrate tolerance to NH_3_ and possess nitrogen-fixing capabilities (57). Meanwhile, the nitrogen utilization mechanism and physiological traits of cyanobacteria could mitigate the impact of free ammonia on microbial communities (58). Cyanobacteria can utilize various nitrogen sources such as NO_3_^-^, NO_2_^-^, and NH_4_^+^ (59). Furthermore, Cyanobacteria exhibit a preference for nitrogen sources in the order NH_4_^+^ > NO_3_^-^ > N_2_ and exclusively utilize NH_4_^+^ when it is available and does not use other nitrogen sources (60). This trait influences the equilibrium of ammonia-nitrogen presence in aquatic environments, leading to a decrease in free ammonia and ammonia-nitrogen concentrations. Furthermore, certain cyanobacteria can thrive even in elevated levels of free ammonia (51, 61). Consequently, this capability may allow certain cyanobacteria to assume the function of the bacteria inhibited by free ammonia, contributing to the stability of the microbial community. These factors could explain why cyanobacteria play a pivotal role in the four networks as critical hubs.

The species extinction was simulated by randomly removing nodes, and the network’s stability was assessed using the average degree and the natural connectivity index. The results indicate that species extinctions seem to have a weaker impact on network stability in networks characterized by low concentrations of free ammonia. This result can be caused by several reasons. First, this study showed that as the concentration of free ammonia increased, the α-diversity of the community decreased aligning with previous descriptions. This reduction also diminishes the functional redundancy of the community to some extent, consequently diminishing the network’s resilience to species extinction (62). Comparable findings were reported by Damian J. Hernandez et al. in their study on environmental stress destabilizing microbial community networks, where they observed lower microbial diversity in habitats under higher stress conditions (55). Second, free ammonia exhibits acute and chronic toxicity toward numerous species, including *Daphnia magna*, *Scophthalmus maximus*, *Lampsilis fasciola*, and others (63–65). Its influence on the entire aquatic food web is top-down, potentially causing fluctuations in the overall aquatic community (66–68). These fluctuations are propagated through the food chain and have the potential to induce alterations in the microbial community structure and a decline in diversity, consequently leading to reduced stability of the microbial community network (69). Third, the increase in ammonia-nitrogen and free ammonia, to some extent, reflects the eutrophication process in water (5, 70). This process frequently results in extensive growth and reproduction of phytoplankton, ultimately leading to the occurrence of algal blooms (71, 72). Moreover, algae blooms were observed at certain sampling sites in this study. This phenomenon leads to competition between algae and bacteria for inorganic nutrients (73). In summary, an elevation in free ammonia levels can give rise to various results, including heightened interspecific competition and diminished diversity, ultimately leading to network instability and decreased robustness (74, 75).

### Assembly process of bacterioplankton community

In order to investigate the assembly process of distinct bacterial subcommunities in response to free ammonia stress, we categorized the processes governing compositional variation within microbial communities into five distinct categories using the ICAMP model, with reference to βNRI and RCbray values (40). The study revealed that both drift and dispersal limitation played dominant roles in shaping the total community, as well as its subcommunities. They obtained similar findings, indicating that marine microbial community assembly is primarily governed by dispersal limitation and drift processes (76). This phenomenon can be caused by several reasons. The high functional redundancy in microbial communities promotes neutrality and increases the susceptibility of functionally redundant populations to drift. The greater abundance of nutrients in aquatic environments creates favorable conditions for the drifting of microbial communities (24, 75). Previous studies have shown that dispersal limitation plays an important role in community assembly, and it can introduce stochasticity by amplifying ecological drift (22, 77). Dispersal limitation, while also a stochastic process, is significantly impacted by environmental conditions. Physical barriers and habitat suitability play crucial roles in determining the success of species dispersal. Environmental heterogeneity across landscapes created a mosaic of suitable and unsuitable habitats, influencing the pattern of dispersal and establishment of species. This phenomenon can lead to a more homogenized community, where only certain taxa can thrive and dominate. This intensifies interspecific competition and leads to the oscillation of environmental factors. Consequently, this may lead to an augmentation of community variability or turnover (21, 78). Furthermore, the results suggest that as the transition is made from the abundant to the rare subcommunity, the impact of drift intensifies, while the influence of dispersal limitation weakens. This pattern is consistent with prior research, underscoring the heightened vulnerability of rare subcommunities within large microbial communities to ecological drift (22). This susceptibility arises from the lower relative abundance of rare communities (79). Environmental factors can influence drift indirectly by affecting population sizes and dynamics. For instance, environmental stressors such as temperature changes or drought can reduce population sizes, making smaller populations more susceptible to stochastic fluctuations and hence more influenced by drift (40). Rare communities struggle to expand their presence numerically compared to members of more abundant communities and are less responsive to environmental changes. Consequently, microorganisms in rare communities may be more susceptible to the adverse effects of drift, such as extinction or failure to survive in a specific environment (80).

### Conclusions

In this study, we employed high throughput 16S rRNA sequencing in conjunction with environmental variables to investigate variations in the composition and diversity of microbial communities across Yuqiao, Panjiakou, and Daheiting reservoirs, their tributaries, and the Luan River basin. Our findings indicate that NH_3_ concentration significantly influences the dynamics of microbial communities, with a notable decrease in community richness and diversity alongside increased dissimilarity under higher NH_3_ conditions. The analysis revealed that certain microbial groups, particularly Actinobacteriaota, were notably more prevalent in environments with elevated NH_3_ levels, suggesting their potential resilience or adaptive responses to NH_3_ stress. Additionally, through co-occurrence network analysis, we observed dynamic changes in network topology and increased connectedness under NH_3_ stress. Key nodes, identified as connectors and module hubs, played crucial roles in maintaining network structure, particularly Cyanobacteria and Actinobacteriaota. Furthermore, stochastic processes, particularly drift and dispersal limitation, predominantly shaped the microbial communities. Within the three subcommunities, the impact of drift became more pronounced as the effect of dispersal limitation diminished.

## Data Availability

All the raw sequencing data were deposited into the NCBI Sequence Read Archive (SRA) database under accession number PRJNA1030205.

## SUPPLEMENTAL MATERIAL

Supplemental figures spectrum.01051-24-s0001.docx Fig. S1 to S4.

Supplemental tables spectrum.01051-24-s0002.docx Tables S1 to S4.

ASM does not own the copyrights to Supplemental Material that may be linked to, or accessed through, an article. The authors have granted ASM a non-exclusive, world-wide license to publish the Supplemental Material files. Please contact the corresponding author directly for reuse.
