# Supplementary material for: Assembly process and co-occurrence network of microbial community in response to free ammonia gradient distribution
Source: Microbiol Spectr. 2024 Jul 26;12(9):e01051-24. doi: 10.1128/spectrum.01051-24 (PMC11370247; doi:10.1128/spectrum.01051-24)
Supplement: Supplemental figures — Fig. S1 to S4. [file spectrum.01051-24-s0001.docx]

**Supplementary Materials Figure**
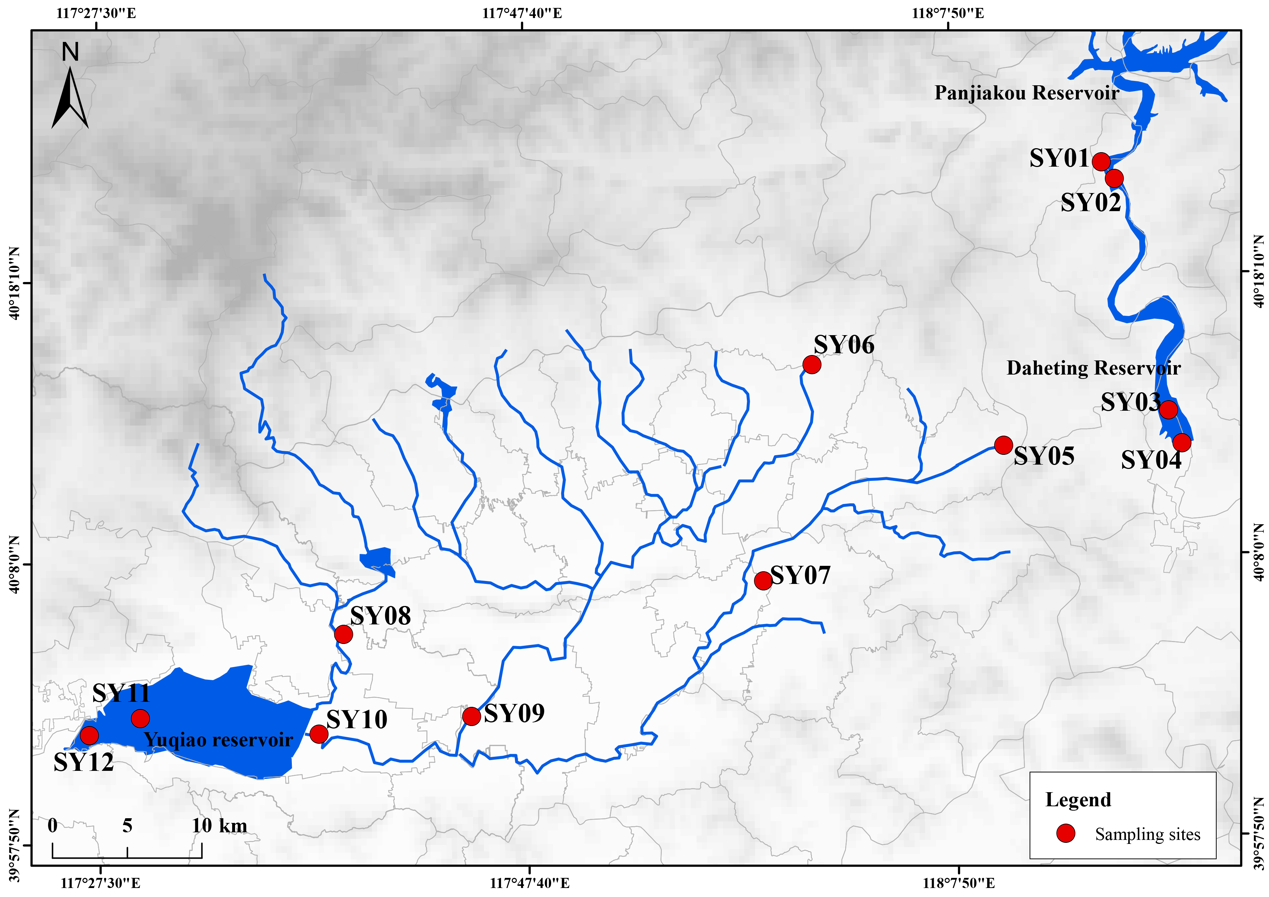


**Fig. S1.** Map of the sampling sites.


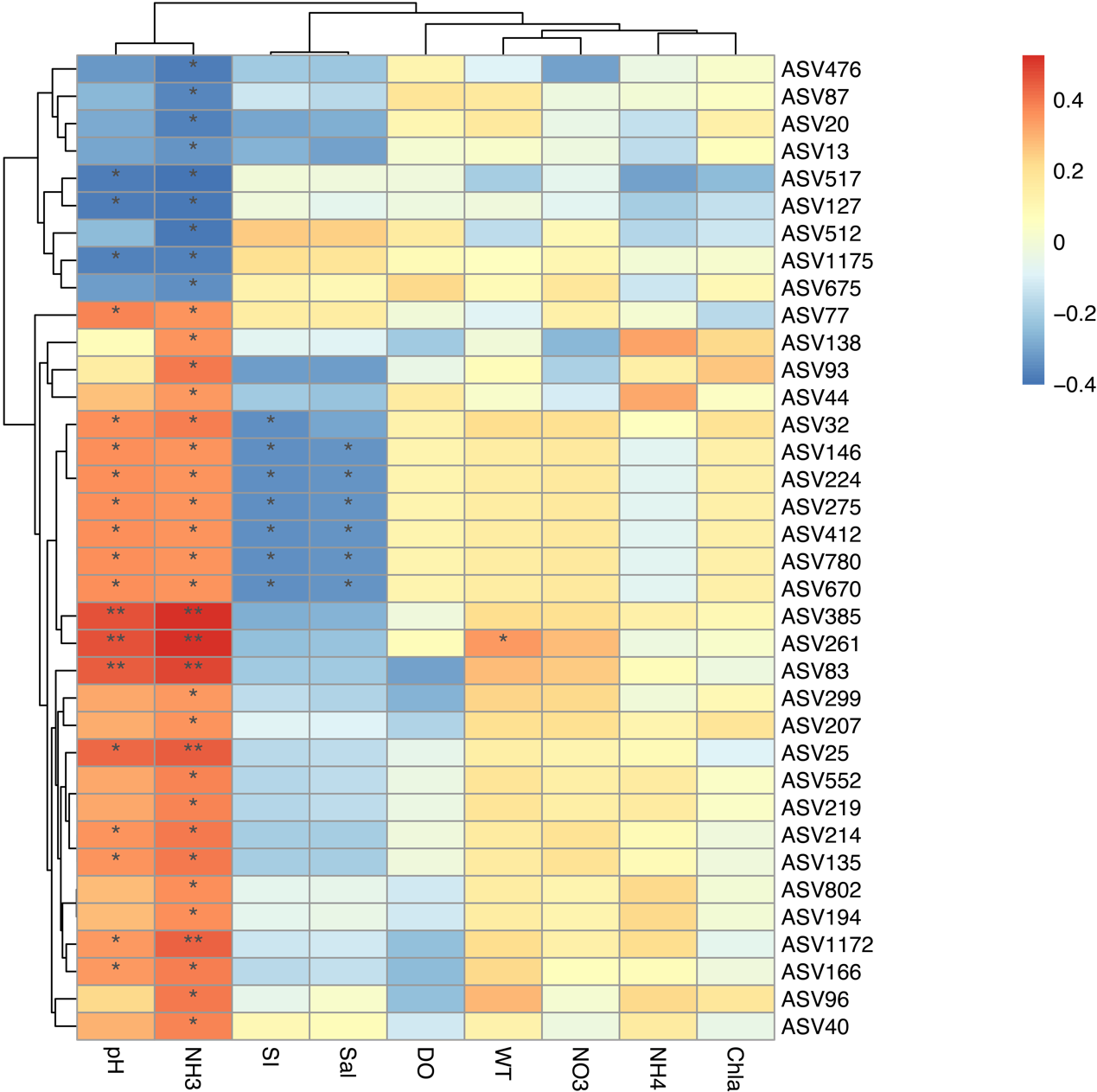


**Fig. S2.** Spearman correlation analysis typical taxa and environmental factors. The *p* value was **p* < 0.05, ***p* < 0.01.


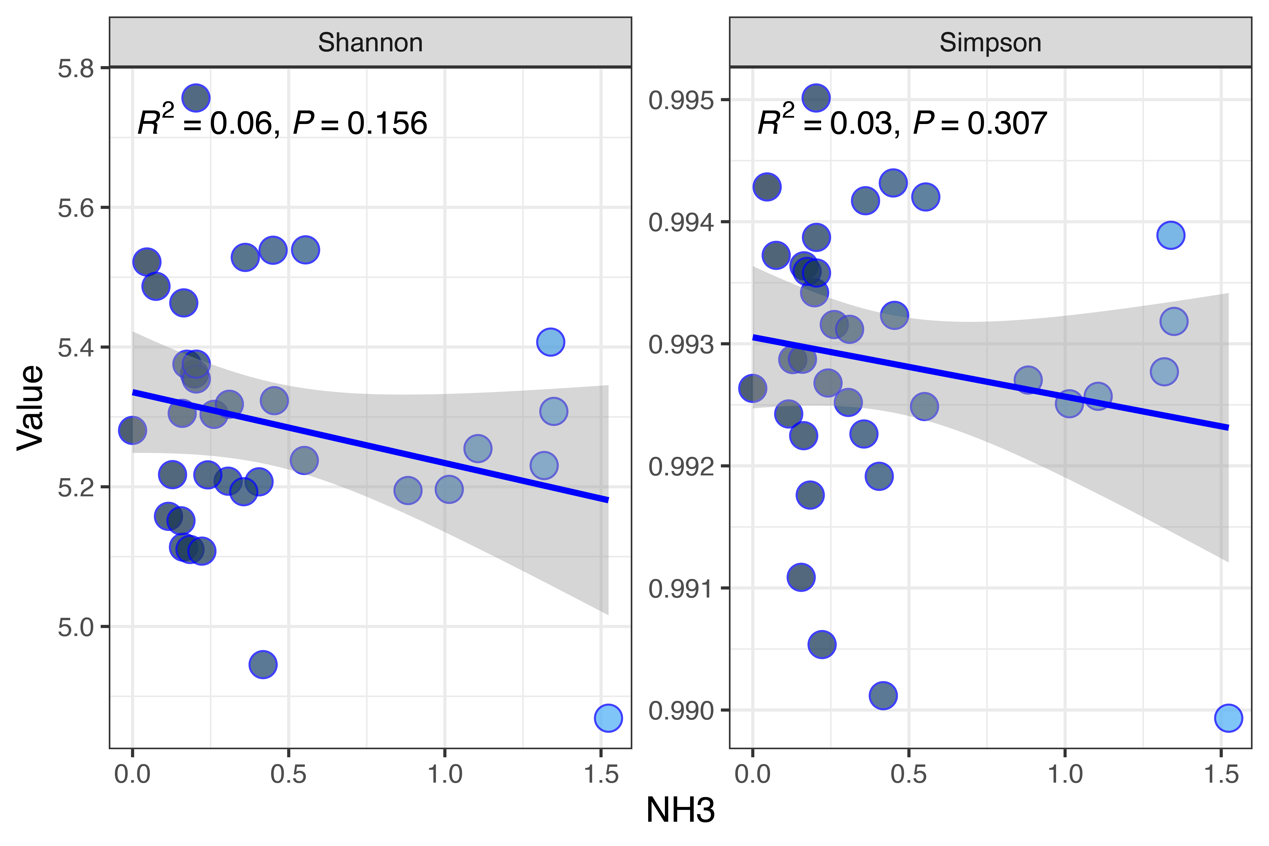


**Fig. S3.** Trends in the Shannon and Simpson of the microbial community with NH3


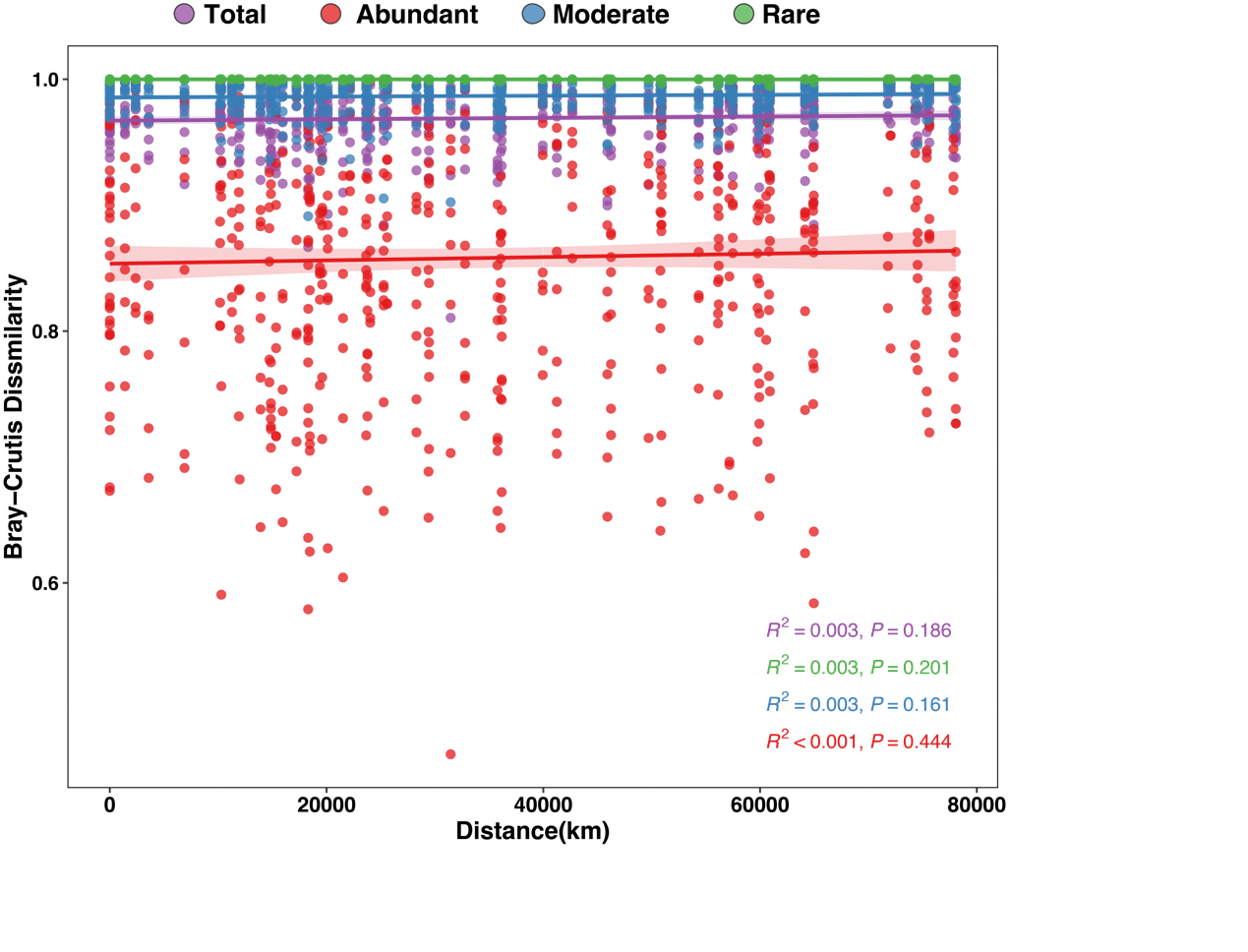


**Fig. S4.** Linear regression between the 'Bray-Curtis' dissimilarity distances of community and the geographic distance.
