# Supplementary material for: Assembly process and co-occurrence network of microbial community in response to free ammonia gradient distribution
Source: Microbiol Spectr. 2024 Jul 26;12(9):e01051-24. doi: 10.1128/spectrum.01051-24 (PMC11370247; doi:10.1128/spectrum.01051-24)
Supplement: Supplemental tables — Tables S1 to S4. [file spectrum.01051-24-s0002.docx]

**Supplementary Materials Table**

**Table S1.** Environmental attributes of the samples

| ID | Latitude  (°N) | Longitude  （°N） | Temperature  (℃) | SI  （mS/cm） | Sal  （‰） | DO  (mg/L) | pH | NH_4_^+^-N  (mg/L) | NH_3_  (mg/L) | NO_3_^-^-N  (mg/L) | Chl  （μg/L） |
| --- | --- | --- | --- | --- | --- | --- | --- | --- | --- | --- | --- |
| SY01_1 | 40.36 | 118.26 | 25.92 | 0.61 | 0.29 | 7.83 | 8.31 | 0.913 | 0.115 | 1.275 | 9.6 |
| SY02_1 | 40.37 | 118.25 | 25.48 | 0.617 | 0.3 | 10.51 | 8.67 | 0.465 | 0.128 | 2.635 | 5.8 |
| SY03_1 | 40.2 | 118.31 | 26.22 | 0.532 | 0.26 | 12.82 | 8.72 | 0.499 | 0.163 | 2.625 | 19 |
| SY04_1 | 40.22 | 118.3 | 26.58 | 0.521 | 0.25 | 13.53 | 8.84 | 0.415 | 0.184 | 3.080 | 15.8 |
| SY05_1 | 40.2 | 118.17 | 17.73 | 0.885 | 0.44 | 15.11 | 8.47 | 0.46 | 0.046 | 2.652 | 5.6 |
| SY06_1 | 40.25 | 118.02 | 26.31 | 0.565 | 0.27 | 11.91 | 8.61 | 0.607 | 0.155 | 2.065 | 16.1 |
| SY07_1 | 40.12 | 117.98 | 22.04 | 1.026 | 0.51 | 8.47 | 8.38 | 0.667 | 0.075 | 1.700 | 6.5 |
| SY08_1 | 40.09 | 117.65 | 19.85 | 0.54 | 0.29 | 10.94 | 9.31 | 0.45 | 0.361 | 3.016 | 47.5 |
| SY09_1 | 40.04 | 117.75 | 22.04 | 0.591 | 0.29 | 21.6 | 9.35 | 0.433 | 0.45 | 2.969 | 3.2 |
| SY10_1 | 40.03 | 117.63 | 26.2 | 0.63 | 0.31 | 11.51 | 8.88 | 1.153 | 0.55 | 1.078 | 8.9 |
| SY11_1 | 40.03 | 117.45 | 0 | 0 | 0 | 0 | 8.5 | 0 | 0.241 | 0 | 12 |
| SY01_2 | 40.36 | 118.26 | 23.58 | 0.589 | 0.29 | 8.88 | 9.44 | 0.921 | 1.318 | 1.910 | 8.1 |
| SY02_2 | 40.37 | 118.25 | 22.78 | 0.58 | 0.28 | 9.31 | 9.56 | 0.763 | 1.349 | 2.499 | 6 |
| SY03_2 | 40.2 | 118.31 | 23.77 | 0.512 | 0.25 | 11.86 | 9.67 | 0.626 | 1.524 | 2.630 | 7.5 |
| SY04_2 | 40.22 | 118.3 | 22.54 | 0.507 | 0.24 | 12.47 | 9.64 | 0.487 | 1.014 | 2.372 | 14.9 |
| SY05_2 | 40.2 | 118.17 | 15.68 | 1.027 | 0.51 | 0 | 9.52 | 0.579 | 0.554 | 3.546 | 0 |
| SY06_2 | 40.25 | 118.02 | 25.3 | 0.516 | 0.25 | 3 | 10.02 | 0.143 | 0.882 | 6.324 | 9.1 |
| SY07_2 | 40.12 | 117.98 | 19.13 | 0.885 | 0.44 | 0.43 | 9.42 | 0.42 | 0.418 | 2.464 | 3.5 |
| SY08_2 | 40.09 | 117.65 | 17.69 | 0.086 | 0 | 0 | 0 | 0 | 0 | 0 | 0 |
| SY09_2 | 40.04 | 117.75 | 21.14 | 0.817 | 0.4 | 4.07 | 9.05 | 0.724 | 0.356 | 1.150 | 4.7 |
| SY10_2 | 40.03 | 117.63 | 22.94 | 0.612 | 0.3 | 1.96 | 9.07 | 0.783 | 0.454 | 1.051 | 13.4 |
| SY11_2 | 40.03 | 117.45 | 23.87 | 0.561 | 0.27 | 1.95 | 9.48 | 0.696 | 1.106 | 2.359 | 4.3 |
| SY12_2 | 40.04 | 117.49 | 24.26 | 0.562 | 0.27 | 4.19 | 9.66 | 0.538 | 1.339 | 3.132 | 19.1 |
| SY01_3 | 40.36 | 118.26 | 19.42 | 0.612 | 0.3 | 10.69 | 9.13 | 0.499 | 0.261 | 1.358 | 3.4 |
| SY02_3 | 40.37 | 118.25 | 19.29 | 0.62 | 0.3 | 9.03 | 9.07 | 0.698 | 0.31 | 1.215 | 3.6 |
| SY03_3 | 40.2 | 118.31 | 17.27 | 0.641 | 0.31 | 12.13 | 9.14 | 0.888 | 0.405 | 1.497 | 3.9 |
| SY04_3 | 40.22 | 118.3 | 17.3 | 0.628 | 0.31 | 13.11 | 9.18 | 0.4 | 0.198 | 1.600 | 3.8 |
| SY05_3 | 40.2 | 118.17 | 17.87 | 0.671 | 0.33 | 13.45 | 8.98 | 0.616 | 0.203 | 1.096 | 25.3 |
| SY06_3 | 40.25 | 118.02 | 20.36 | 0.557 | 0.27 | 11.43 | 9.13 | 0.546 | 0.306 | 1.274 | 3.2 |
| SY07_3 | 40.12 | 117.98 | 16.19 | 0.832 | 0.41 | 16.07 | 8.97 | 0.79 | 0.222 | 1.100 | 111.7 |
| SY08_3 | 40.09 | 117.65 | 15.68 | 0.509 | 0.25 | 16.3 | 8.79 | 0.922 | 0.164 | 0.760 | 3.5 |
| SY09_3 | 40.04 | 117.75 | 15.83 | 0.727 | 0.36 | 13.65 | 8.77 | 0.921 | 0.159 | 0.836 | 1.8 |
| SY10_3 | 40.03 | 117.63 | 16.85 | 0.595 | 0.29 | 10.66 | 8.78 | 0.918 | 0.174 | 0.796 | 7.1 |
| SY11_3 | 40.03 | 117.45 | 17.86 | 0.583 | 0.28 | 13.89 | 8.82 | 1.301 | 0.204 | 0.776 | 9.3 |
| SY12_3 | 40.04 | 117.49 | 17.75 | 0.583 | 0.28 | 13.7 | 8.74 | 1.804 | 0.204 | 0.670 | 11.6 |

**Table S2.** Network Topology Indices

| Network Topology Indices | n1 | n2 | n3 | n4 |
| --- | --- | --- | --- | --- |
| Total nodes | 250.00 | 266.00 | 269.00 | 260.00 |
| Total links | 342.00 | 365.00 | 352.00 | 354.00 |
| R square of power-law | 0.62 | 0.45 | 0.57 | 0.63 |
| Average degree (avgK) | 2.74 | 2.74 | 2.62 | 2.72 |
| Average clustering coefficient (avgCC) | 0.09 | 0.09 | 0.07 | 0.08 |
| Average path distance (GD) | 8.04 | 12.33 | 11.49 | 9.02 |
| Geodesic efficiency (E) | 0.19 | 0.13 | 0.13 | 0.15 |
| Harmonic geodesic distance (HD) | 5.15 | 7.92 | 7.90 | 6.78 |
| Maximal degree | 8.00 | 7.00 | 8.00 | 8.00 |
| Centralization of degree (CD) | 0.02 | 0.02 | 0.02 | 0.02 |
| Maximal betweenness | 3607.08 | 8580.64 | 8525.87 | 7486.59 |
| Nodes with max betweenness | ASV163 | ASV | ASV50 | ASV3 |
| Centralization of betweenness (CB) | 0.11 | 0.21 | 0.21 | 0.20 |
| Maximal stress centrality | 7250.00 | 43095.00 | 53156.00 | 23504.00 |
| Centralization of stress centrality (CS) | 0.22 | 1.10 | 1.34 | 0.63 |
| Nodes with max eigenvector centrality | ASV100 | ASV128 | ASV205 | ASV118 |
| Centralization of eigenvector centrality (CE) | 0.96 | 0.97 | 0.97 | 0.97 |
| Maximal closeness centrality | 0.00 | 0.00 | 0.00 | 0.00 |
| Centralization of closeness centrality (CCL) | 0.00 | 0.01 | 0.01 | 0.01 |
| Density (D) | 0.01 | 0.01 | 0.01 | 0.01 |
| Transitivity (Trans) | 0.10 | 0.09 | 0.08 | 0.09 |
| Connectedness (Con) | 0.33 | 0.75 | 0.78 | 0.83 |
| Efficiency | 0.98 | 0.99 | 0.99 | 0.99 |

**Table S3.** The nodes properties of networks

| Nodes | Within Module Connectivity | Among Module Connectivity | Degree | Modularity | Community | Type | Network |
| --- | --- | --- | --- | --- | --- | --- | --- |
| ASV100 | 2.13 | 0.00 | 7 | 1 | intermediate | Module hubs | A |
| ASV109 | 2.09 | 0.28 | 6 | 9 | intermediate | Module hubs | A |
| ASV164 | 2.09 | 0.00 | 4 | 7 | intermediate | Module hubs | A |
| ASV196 | 2.19 | 0.00 | 6 | 12 | intermediate | Module hubs | A |
| ASV2 | 2.17 | 0.00 | 5 | 2 | abundant | Module hubs | A |
| ASV39 | 2.17 | 0.00 | 5 | 2 | abundant | Module hubs | A |
| ASV65 | 3.04 | 0.00 | 8 | 11 | abundant | Module hubs | A |
| ASV168 | 2.41 | 0.00 | 6 | 7 | intermediate | Module hubs | B |
| ASV169 | 2.16 | 0.00 | 6 | 11 | intermediate | Module hubs | B |
| ASV211 | 2.07 | 0.00 | 7 | 14 | intermediate | Module hubs | B |
| ASV28 | 3.01 | 0.00 | 7 | 4 | abundant | Module hubs | B |
| ASV394 | 2.76 | 0.00 | 7 | 3 | intermediate | Module hubs | B |
| ASV441 | 2.35 | 0.00 | 4 | 1 | intermediate | Module hubs | B |
| ASV48 | 2.16 | 0.00 | 6 | 11 | abundant | Module hubs | B |
| ASV5 | -1.53 | 0.67 | 3 | 8 | abundant | Connectors | B |
| ASV50 | 2.57 | 0.00 | 5 | 10 | abundant | Module hubs | B |
| ASV62 | 2.16 | 0.00 | 6 | 11 | abundant | Module hubs | B |
| ASV98 | -0.43 | 0.63 | 4 | 4 | intermediate | Connectors | B |
| ASV201 | 2.41 | 0.00 | 5 | 14 | intermediate | Module hubs | C |
| ASV205 | 2.21 | 0.00 | 8 | 3 | intermediate | Module hubs | C |
| ASV228 | -0.49 | 0.63 | 4 | 13 | intermediate | Connectors | C |
| ASV26 | 2.58 | 0.00 | 6 | 6 | abundant | Module hubs | C |
| ASV51 | 2.00 | 0.28 | 6 | 15 | abundant | Module hubs | C |
| ASV57 | 2.87 | 0.24 | 7 | 5 | abundant | Module hubs | C |
| ASV61 | 2.56 | 0.00 | 5 | 4 | abundant | Module hubs | C |
| ASV106 | 0.19 | 0.63 | 4 | 5 | intermediate | Connectors | D |
| ASV113 | 2.90 | 0.22 | 8 | 8 | intermediate | Module hubs | D |
| ASV15 | 2.35 | 0.28 | 6 | 2 | abundant | Module hubs | D |
| ASV171 | 2.42 | 0.00 | 6 | 11 | intermediate | Module hubs | D |
| ASV18 | 2.15 | 0.00 | 4 | 10 | abundant | Module hubs | D |
| ASV239 | 2.78 | 0.32 | 5 | 5 | intermediate | Module hubs | D |
| ASV282 | 2.81 | 0.00 | 7 | 14 | intermediate | Module hubs | D |
| ASV287 | 0.91 | 0.61 | 7 | 11 | intermediate | Connectors | D |
| ASV3 | -1.36 | 0.67 | 3 | 11 | abundant | Connectors | D |
| ASV34 | 2.14 | 0.00 | 6 | 3 | abundant | Module hubs | D |
| ASV534 | 2.25 | 0.00 | 5 | 1 | intermediate | Module hubs | D |
| ASV63 | -1.21 | 0.67 | 3 | 1 | abundant | Connectors | D |
| ASV859 | 2.15 | 0.28 | 6 | 4 | intermediate | Module hubs | D |

**Table S4.** Network stability was evaluated based on average degree and natural connectivity following species extinction by proportionally removing nodes at random.

| remove node | type | values | network |
| --- | --- | --- | --- |
| 0 | average degree | 2.74 | n1 |
| 5% | average degree | 2.66 | n1 |
| 10% | average degree | 2.51 | n1 |
| 15% | average degree | 2.40 | n1 |
| 20% | average degree | 2.28 | n1 |
| 25% | average degree | 2.18 | n1 |
| 30% | average degree | 1.99 | n1 |
| 35% | average degree | 1.90 | n1 |
| 40% | average degree | 1.72 | n1 |
| 45% | average degree | 1.51 | n1 |
| 50% | average degree | 1.52 | n1 |
| 0 | average degree | 2.74 | n2 |
| 5% | average degree | 2.59 | n2 |
| 10% | average degree | 2.45 | n2 |
| 15% | average degree | 2.32 | n2 |
| 20% | average degree | 2.17 | n2 |
| 25% | average degree | 2.13 | n2 |
| 30% | average degree | 2.08 | n2 |
| 35% | average degree | 1.80 | n2 |
| 40% | average degree | 1.59 | n2 |
| 45% | average degree | 1.55 | n2 |
| 50% | average degree | 1.35 | n2 |
| 0 | average degree | 2.62 | n3 |
| 5% | average degree | 2.42 | n3 |
| 10% | average degree | 2.36 | n3 |
| 15% | average degree | 2.26 | n3 |
| 20% | average degree | 2.16 | n3 |
| 25% | average degree | 1.98 | n3 |
| 30% | average degree | 1.63 | n3 |
| 35% | average degree | 1.74 | n3 |
| 40% | average degree | 1.69 | n3 |
| 45% | average degree | 1.34 | n3 |
| 50% | average degree | 1.38 | n3 |
| 0 | average degree | 2.72 | n4 |
| 5% | average degree | 2.61 | n4 |
| 10% | average degree | 2.40 | n4 |
| 15% | average degree | 2.34 | n4 |
| 20% | average degree | 2.21 | n4 |
| 25% | average degree | 2.00 | n4 |
| 30% | average degree | 1.87 | n4 |
| 35% | average degree | 1.65 | n4 |
| 40% | average degree | 1.63 | n4 |
| 45% | average degree | 1.49 | n4 |
| 50% | average degree | 1.44 | n4 |
| 0 | natural connectivity | 1.35 | n1 |
| 5% | natural connectivity | 1.31 | n1 |
| 10% | natural connectivity | 1.23 | n1 |
| 15% | natural connectivity | 1.19 | n1 |
| 20% | natural connectivity | 1.13 | n1 |
| 25% | natural connectivity | 1.08 | n1 |
| 30% | natural connectivity | 0.98 | n1 |
| 35% | natural connectivity | 0.95 | n1 |
| 40% | natural connectivity | 0.85 | n1 |
| 45% | natural connectivity | 0.76 | n1 |
| 50% | natural connectivity | 0.76 | n1 |
| 0 | natural connectivity | 1.33 | n2 |
| 5% | natural connectivity | 1.26 | n2 |
| 10% | natural connectivity | 1.19 | n2 |
| 15% | natural connectivity | 1.14 | n2 |
| 20% | natural connectivity | 1.07 | n2 |
| 25% | natural connectivity | 1.06 | n2 |
| 30% | natural connectivity | 1.00 | n2 |
| 35% | natural connectivity | 0.83 | n2 |
| 40% | natural connectivity | 0.74 | n2 |
| 45% | natural connectivity | 0.73 | n2 |
| 50% | natural connectivity | 0.67 | n2 |
| 0 | natural connectivity | 1.28 | n3 |
| 5% | natural connectivity | 1.15 | n3 |
| 10% | natural connectivity | 1.15 | n3 |
| 15% | natural connectivity | 1.09 | n3 |
| 20% | natural connectivity | 1.06 | n3 |
| 25% | natural connectivity | 0.98 | n3 |
| 30% | natural connectivity | 0.79 | n3 |
| 35% | natural connectivity | 0.86 | n3 |
| 40% | natural connectivity | 0.82 | n3 |
| 45% | natural connectivity | 0.66 | n3 |
| 50% | natural connectivity | 0.69 | n3 |
| 0 | natural connectivity | 1.36 | n4 |
| 5% | natural connectivity | 1.31 | n4 |
| 10% | natural connectivity | 1.13 | n4 |
| 15% | natural connectivity | 1.14 | n4 |
| 20% | natural connectivity | 1.08 | n4 |
| 25% | natural connectivity | 1.03 | n4 |
| 30% | natural connectivity | 0.89 | n4 |
| 35% | natural connectivity | 0.84 | n4 |
| 40% | natural connectivity | 0.84 | n4 |
| 45% | natural connectivity | 0.72 | n4 |
| 50% | natural connectivity | 0.76 | n4 |
